# The Preliminary Exploration of Micro-Friction Stir Welding Process and Material Flow of Copper and Brass Ultra-Thin Sheets

**DOI:** 10.3390/ma13102401

**Published:** 2020-05-22

**Authors:** Changqing Zhang, Zhuo Qin, Chen Rong, Wenchen Shi, Shuwen Wang

**Affiliations:** 1 State Key Laboratory of Advanced Processing and Recycling of Non-ferrous Metal, Lanzhou University of Technology, Lanzhou 730050, China; 2 School of Material Science and Engineering, Lanzhou University of Technology, Lanzhou 730050, China; qzqyijsvsci@163.com (Z.Q.); 13893104421@163.com (C.R.); shiwcswc@163.com (W.S.); welding_wsw@163.com (S.W.)

**Keywords:** μFSW, copper, brass, dissimilar ultra-thin sheet, welding parameters, material flow

## Abstract

In the friction stir welding (FSW) of ultra-thin dissimilar metal sheets, different physical material properties, the reduction of plastic metal in the weld zone, and insufficient plastic metal flow lead to poor weld seam shapes and joint qualities. Therefore, it is necessary to study the flow behavior during the FSW of ultrathin sheets. In this study, micro friction stir welding (μFSW) was conducted and analyzed for the butt welding of 0.6-mm-thick ultrathin brass (H62-H) and pure copper (T2-Y) sheets. By analyzing the electric signals of the temperature and force during the welding process, testing the mechanical properties, and analyzing the metallography of the joint, the influences of the process parameters on the metal flow behavior during μFSW were studied. In the proper process conditions, the material preferentially migrated and concentric vortex flow occurred in the vicinity of the shoulder and tool pin action areas. The copper was pushed from the retreating side (RS) to the advancing side (AS) of the weld, allowing it to flow more fully. A mixture of both materials formed at the bottom of the weld nugget, and less migration occurred in the heat-affected zone of the AS at this time. The highest tensile strength can reach 194 MPa, accounting for 82.6% of the copper. The presence of brittle phases Cu_5_Zn_8_, AgZn_3_ and AgZn caused the hardness to fluctuate slightly.

## 1. Introduction

With the rapid development of aviation, aerospace, electronics, energy, and other industrial fields, product components are becoming increasingly miniaturized, and the demand for micro-connection technology is also increasing. Copper and its alloys are the preferred materials for lightweight, miniaturized, precision, intelligent and complex processes owing to their unique properties, such as their good electrical conductivities, thermal conductivities, ductilities, and corrosion resistance [1,2]. Friction stir welding (FSW) is an advanced solid-state joining technology that was invented by The Welding Institute (TWI) in 1991. Micro-friction stir welding (μFSW) for sheets with thicknesses less than 1 mm was developed based on traditional FSW, and it is typically a high rotation speeds process [3,4]. The joint possesses a narrow welding seam, and the heat-affected zone exhibits small deformation, However, problems occur when welding thin plates, which include the following: the parent material are easily tears, weld forming is difficult, and the inadequate joint strength creates difficulties for achieving clamping precision and the other precision requirements. There have been several reports on aluminum and its alloys [5,6,7]. For copper and its alloys, in addition to the difficulties mentioned above, because of its generally high melting points and thermal conductivities it is more difficult for the materials to soften and reach plastic states. Consequently, the welds are prone to defects such as voids, incompletely filled groove, and flash. Li et al. found that the mixing of dissimilar materials and the microstructure formation of joints closely depend on the flow of materials [8]. Studying the plastic flow behavior of the joint metal is useful to understand the formation of weld defects, the evolution of the microstructure, to optimize the welding parameters, and to provide a theoretical basis for obtaining well-formed and high-quality joints. 

With the combined thermo-mechanical action, the metal in the weld zone softens and reaches a plastic state, the plastic metal forms a plastic flow shape under the driving force of the tool, and finally, the metal forms a dense weld by metallurgical combination. For ultra-thin plate materials, the metal flow is inadequate due to the reduction in the size of the welding tool. The amount of plastic metal in the weld zone is reduced, and the amount of heat generation is reduced due to the increase in the specific surface area. In response to this problem, scholars have adopted a processing method involving high rotation speeds, low welding speeds, and high plunge depths to increase the line energy and the temperature of the welding zone. Liu et al. used high-rotation-speed FSW technology (8000 rpm) to achieve a butt joint with a 0.8-mm thickness using 6061-T6 aluminum alloy sheets. Compared with the conventional rotation speed (2000 rpm), the heat input increased significantly, and the area of material flow becomes larger, which effectively prevented weak connection defects [9]. Huang et al. studied the weld morphology by changing the plunge depth of the tool using 0.5-mm-thick 6061-T4 sheets. When the plunge depth was 0.02 mm, the heat input was insufficient, and furrow-like defects formed on the advancing side (AS) of the weld. When the plunge depth was 0.05 mm, a relatively complete joint could be obtained. However, when it continued to increase, flash occurred on the retreating side (RS) [10]. Seidel et al. studied the flow topography of the AA2195-T8 material under different process parameters using marker insertion technology (MIT), and heat input increased with the increase in the ratio of the rotating speed to the welding speed, as did the migration quantity of the plasticized metal per unit length of the weld [11,12]. In addition to changing the welding parameters to increase the heat input, the shape and size of the tool are also important factors for driving the flow of material fully. Simoncini et al. compared the influence of two groups of tools with different shoulder diameters on a 1-mm-thick AA5754 aluminum alloy joint and found that using a large shoulder was more conducive to obtaining a good joint [13]. Leal et al. discussed the influence of the shoulder shape on the microstructure and mechanical properties of the joint. The formability and flow of a joint with a concave shoulder shaped were significantly higher than those obtained with a threaded tool [14]. Padmanaban et al. found that a threaded pin increased the force of the material flow in the weld, which resulted in a high-quality weld with better mechanical properties [15]. For ultra-thin sheets, because the size of the pin was small and the shape was difficult to process, the geometric gap of the pin was easily filled, and the influence on the flow of the metal was weakened.

In recent years, scholars have obtained various results on the material flow of FSW welds in thick plates using experiments or simulations. One of the obstacles to a better understanding of the actual flow regimes is the need to visualise the details of the flow features for the cross section of the weld [16,17,18,19,20,21]. Lower rotation and welding speeds are often used in welding plates. The material temperature gradient in the thickness direction of the joint is large, and different thermomechanical processes occur in different locations [22,23]. Ji et al. found that the plasticized material on the surface layer of the weld migrated horizontally under the action of the shoulder. The plastic material in the middle of the nugget area moved upward under the extrusion of the bottom metal, and the bottom of the weld was mainly squeezed, resulting in a poor fluidity of the material [24]. Shah et al. studied dissimilar friction stir welding of AA5052-AA6061 aluminum alloys with varying tool offsets. They found that the tool offset plays a major role in the stir zone material flow, and zero tool offset provided the optimal intermixing for this alloy combination [25]. Mugada et al. worked the tool geometry such as shoulder and pin designs to play a significant role in altering the material flow characteristics. They identified that for each shoulder there was a compatible pin shape for complete shearing and distribution of plasticized material in the weld zone [26,27,28,29,30]. For ultra-thin sheet μFSW, the temperature gradient between the upper and lower layers of the weld is small, and the effect of the shoulder on the weldment extrusion and metal flow is strengthened. The weld formation, material heat production, and flow mechanism are different from those of conventional FSW, so further research is needed. In this paper, by analyzing the temperature, axial force, transverse force, material migration, microstructural transformation and mechanical properties in μFSW, the factors that influence the weld material flow behavior are thoroughly studied, and the formation mechanism of friction stir-welding joints of ultra-thin plates of copper and its alloys is analyzed. 

## 2. Materials and Methods

**Figure 1:**
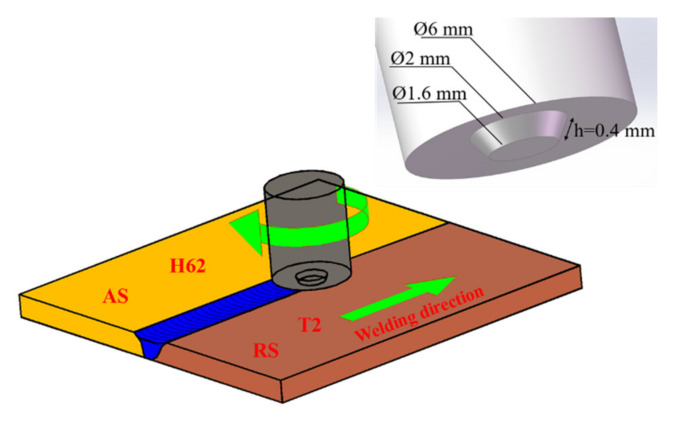
Schematic of micro-friction stir welding (μFSW) and the tool. (AS—advancing side of the weld-brass-H62; RS—retreating side of the weld-copper-T2).

In the experiment, brass (H62) and copper (T2) sheet specimens with 0.6 mm thicknesses were prepared. The main chemical components and physical properties are provided in Table 1 and Table 2, respectively. The material sheets were sheared into 100 × 25 mm coupons as test workpieces, and the surfaces of the sheets were cleaned with acetone before welding to remove oil and debris. The tool was made of WC-Co (The content of tungsten carbide in the WC-Co hardmetals was 85 94 wt.% (76.4 90.0 vol.%)) cemented carbide [31], which was composed of a 3° concave shoulder and a conical pin. The tool had a 6-mm shoulder diameter, and the pin was tapered from 2 mm at the root diameter to 1.6 mm at the tip diameter, with a length of 0.4 mm. In the welding process, the temperature of the AS of the weld was higher than that of the RS [32]. A marked improvement in the surface appearance was obtained by placing brass in the AS and copper on the RS. The self-developed μFSW machine was used to carry out butt joint experiments on the sheets. The rotation speed adjustment range was 10,000–14,000 rpm, and the welding speed adjustment range was 160–320 mm/min. The parameter matching could be adjusted within this range. A schematic of the μFSW machine and the tool is shown in Figure 1.

**Figure 2:**
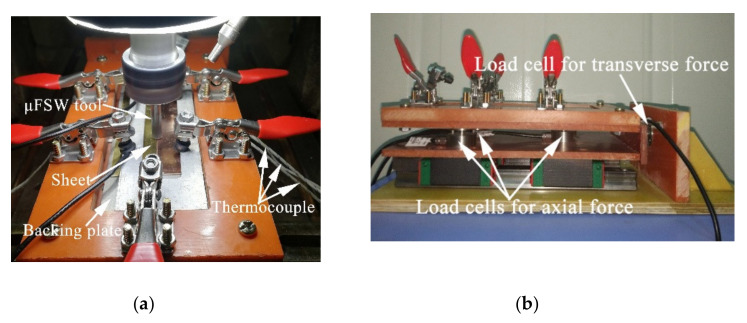

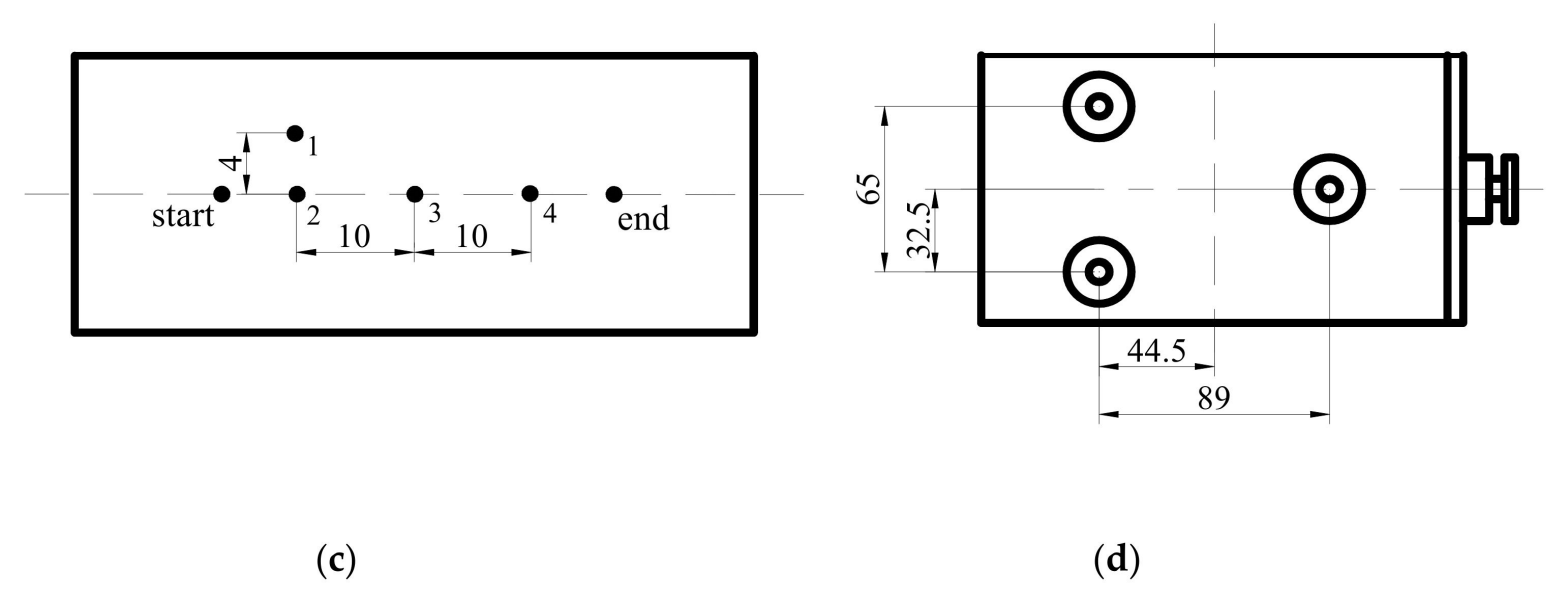
Welding temperature and welding force measuring device and distribution map: (**a**,**b**) physical diagram; (**c**,**d**) distribution map.

The temperature, axial force, and transverse force under various welding parameters were measured by a custom-made device. To reduce the heat loss, a titanium alloy with a lower thermal conductivity was selected for the backing sheets. The probe of the k-type thermocouple was fixed in the middle area of the backing sheets and the bottom of the weld with high temperature glue, which was more convenient for measuring the temperature of the weld. Point 1 was located 4 cm from point 2 on the AS, and points 2–4 were located at the bottom of the weld center (Figure 2a,c). The axial force was measured by three load cells fixed along the *Z*-axis (tool direction), and the transverse force was measured by one load cell fixed on the *X*-axis (welding direction) (Figure 2b,d). The signal acquisition was recorded through the NI (National Instruments Corporation, Austin, TX, USA) USB-6008 data acquisition card and the LabView 13.0 software (National Instruments Corporation, Austin, TX, USA). The sampling rate was 1000 Hz, and the collected data were smoothed using a 500 pts Savitzky–Golay smooth, filter to make the variation trend of the curve clearer. The feeding rate of the initial tool was set to a small value. The dwell time the at start of the weld was 6.5 s after the plunge depth reached 0.06 mm, and the weld length was 30 mm. Finally, the tool stayed on the welding seam for 2 s and is drawn out to form a complete welding seam. The welding parameters were listed in Table 3. Under each set of welding parameters, welding tests were performed with the tools for three times.

The specimens were cut from the welded sheets normal to the welding direction. After metallographic and mechanical polishing, the samples were cleaned with a solution (10 g FeCl_3_, 6 mL HCl, 40 mL H_2_O, and 60 mL C_2_H_5_OH). The flow patterns of areas with different weld plasticities were observed by optical microscopy Axio Scope A1 (Zeiss Corporation, Oberkochen, Baden-Württemberg, Germany), and the phase of the cross interface of the joint was analyzed using a D8 advance X-ray diffractometer (Bruker Group, Karlsruhe, Germany). The Vickers microhardness of the joint was measured using a W1102D37 Hardness Tester (Bella Corporation, Lake Bluff, IL, USA). The top layer was 0.2 mm above the top surface, and the bottom layer was 0.2 mm below the bottom surface. The points were separated by 0.2 mm. The load was 0.98 N for 15 s at each point. Tensile tests of the joint were carried out at room temperature using a Instron 3382 universal testing machine (Instron Corporation, Norwood, MA, USA). The speed was set to 0.5 mm/min, and the fracture surfaces were observed on a FEG-450 SEM thermal field emission scanning electron microscope (FEI Corporation, Hillsboro, OR, USA).

## 3. Results and Discussion

### 3.1. Joint Surface Morphology

**Figure 3:**
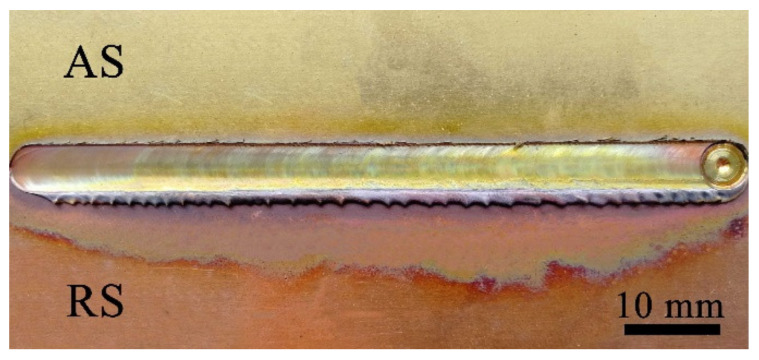
The surface appearance of H62/T2 joints.

The surface morphologies of the joints at a high rotation speed (12,000 rpm) are shown in Figure 3. The welds had well-formed surfaces overall without any grooves and cracks, and the flash was small. Due to the high heat input per unit length of the weld, the color corresponding to oxidation was evident. The oxidation and softening degree of copper (RS) was higher than that of brass (AS). A well-formed weld could be obtained by optimizing the process parameters.

### 3.2. Influence of Process Parameters on Metal Fluidity

**Figure 4:**
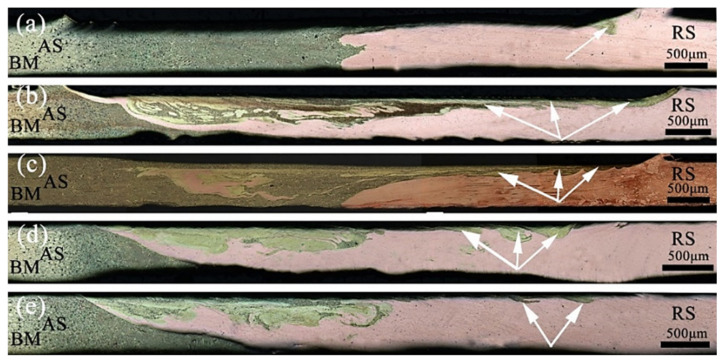
Micromorphology of H62/T2 joints at different welding speeds of 12,000 rpm: (**a**) 320 mm/min, (**b**) 280 mm/min, (**c**) 240 mm/min, (**d**) 200 mm/min, (**e**) 160 mm/min.

The effect of the welding speed on the metal flow was examined. Figure 4 shows the cross-sectional macroscopic morphology of the joints with a fixed rotation speed of 12,000 rpm and a welding speed reduction from 320 to 160 mm/min. Kolnes et al. reported that heat input and strain rate increase with decreasing welding speed, because of the increasing degree of dynamic recrystallization [33]. When the welding speed was 320 mm/min, the volume of the metal being stirred increased per unit length, and the amount of friction decreased correspondingly. As the heat input increases, the metal plasticity decreased, the restraint effect on the expansion of the nugget area increased, and the extrusion pressure on the metal at the bottom of the weld decreased. The flow of plasticized metal was not sufficient, and some materials at the interface simply expanded, which did not generate circular motion with the rotation of the pin, creating a zigzag-shaped interface (Figure 4a). With the decrease in the welding speed, the frictional heat generation increased, and the temperature increased. The interlayer flow was more likely to occur when the metal to be welded reached a plastic state. Because the linear speed of the shoulder of the tool was greater than that of the pin, the brass on the forward side more easily migrated to the RS under the action of the shoulder. The bottom metal flowed forward to the upper end of the AS under the influence of the extrusion of the tool and the hindrance of the backing plate. The copper in the vicinity of the pin and the bottom area increased to the forward side. In the process of plastic flow, the two metals in the nugget zone were intertwined and diffused into each other. At this time, the mechanical properties were higher (Figure 4b). The plastic metal in the nugget zone of the joint had good fluidity. There was resistance to the plastic metal on the RS, which caused the material to flow upward, causing a small amount of flash on the surface of the joint (Figure 4c). When the welding speed continued to decrease, the weld temperature was inversely proportional to the welding speed. The degree of plasticization was too high, and the viscosity decreased sharply, which was not conducive to the full flow of the metal driven by the tool. Thus the metal migration was correspondingly reduced (Figure 4d,e).

When the rotation speed was 12,000 rpm, with the increase in the ratio of the tool rotation speed to the welding speed, the migration of the metal on the AS of the upper layer of the weld cross-section to the RS increased first and then decreased, and the mixing degree of the two metals in the center area also changed like this. Overall, the migration of copper on the RS of the weld was greater than that on the AS. The reason was that the tool automatically leaned toward the softer side of the material during the welding process. The flow capacity of the plasticized metal was enhanced under the action of stirring, driving, and extrusion of the tool. In addition, the difference in the flow speed between the two metals in the nugget zone caused the plastic flow capacity of brass to be less than that of copper in the process of mutual flow.

**Figure 5:**
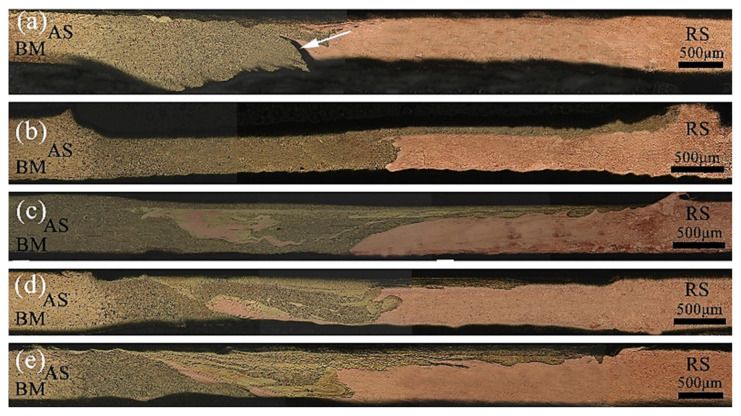
Micromorphology of H62/T2 joints under different welding speeds of 240 mm/min: (**a**) 10,000 rpm, (**b**) 11,000 rpm, (**c**) 12,000 rpm, (**d**) 13,000 rpm, (**e**) 14,000 rpm.

The effect of the rotation speed on the metal flow was examined. Figure 5 shows the cross-sectional macroscopic morphologies of the joints with a fixed welding speed of 240 mm/min and an increasing rotation speed from 10,000 to 14,000 rpm. When the rotation speed was 10,000 rpm, the frictional heat generation per unit time was lower, and the temperature at the shoulder and the bottom of the pin was relatively low. The inherent yield shear stress of the dissimilar material, the degree of softening, and the plastic state of the metal were significantly different. When the metal on both sides reached the middle area of the weld, the driving force of the dynamic recrystallization decreased rapidly, and thus, the plastic metal fluidity was relatively poor. Only small amounts of the metals at the joint interpenetrated each other, with unwelded defects appearing near the bottom of the weld (Figure 5a). With the increase in the rotation speed, the heat input at the weld, the amount of plastic metal, and the viscosity of the metal decreased. Furthermore, the material flow around the tool become more fluid, the shoulder area generated heat and the linear velocity was large, Consequently, the nearby metal preferentially flowed from the AS to the RS, and the penetration of the two metals in the stirring area of the tool increased (Figure 5b). The migration of the brass on the AS of the weld shoulder to the RS increased, and the plastic metal fluidity in the middle area of the pin was correspondingly enhanced. The dissimilar metals migrated into each other to achieve a metallurgical combination, and the material in the bottom area flowed from the RS to the AS. As a result, the mechanical properties increased. (Figure 5c), When the rotation speed was increased again, the temperature at the weld joint was too high. Thus the metal material was more easily softened, and the degree of softening increased. The flow capacity of the metal deteriorated, and the amount of the metal in the bottom area flowing from the RS to the AS decreased (Figure 5d,e). When the welding speed was constant but the rotation speed was varied, the metal near the shoulder of the tool changed from no migration to mutual flow with the increase in the rotation speed, and the lack of welding at the bottom became a dense weld. Thus the plastic metal flowed more fully. The plastic flow speed decreased with the increase in the rotation speed.

### 3.3. Tensile Properties of Joint and Fracture Morphology

**Figure 6:**
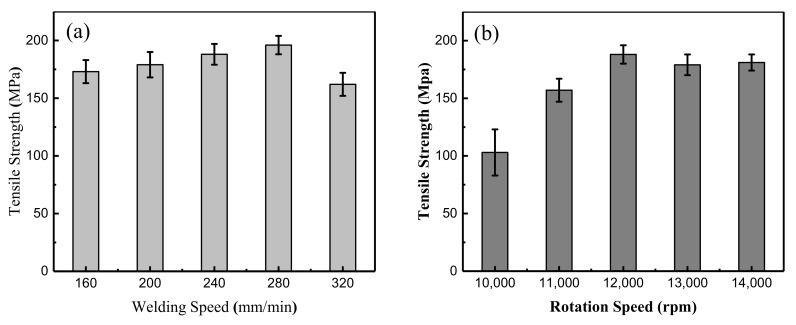
Tensile strength of T2/H62 joint under different process parameters: (**a**) different welding speeds; (**b**) different rotation speeds.

The mechanical properties were the result of a combination of the material flow and various welding defects. Figure 6 shows the average tensile strength of the μFSW butt joints under different process parameters. The results suggested that the joint strength underwent a regular change under the effect of the speed field. As shown in Figure 6a, when the welding speed is fixed, the tensile strength increases first and then decreases with the growth of rotation speed. When the rotation speed was 12,000 rpm and the welding speed was 280 mm/min, the tensile strength of the joint reached the highest value of 194 MPa (82.6% of that of the T2 base metal). At this time, the material flow shape in the weld was optimal. Figure 6b reflects two important regular features of the strength variation at different rotation speeds. Firstly, when the rotation speed is fixed, the tensile strength increases first and then decreases with the growth of the welding speed. Secondly, when the rotation speed of the tool is less than 10,000 rpm, there is insufficient heat input, resulting in a poor metal fluidity in the weld and weak connection between two test sheets, the mechanical properties of weld defects decreased rapidly.

**Figure 7:**
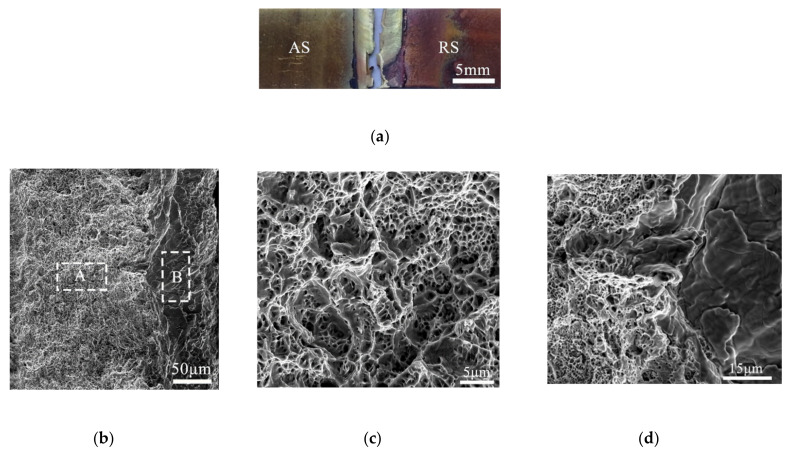
Macro and microfracture surface morphologies of joints (12,000 rpm, 280 mm/min): (**a**) Fracture modes of the joint; (**b**) Fracture surface morphologies of the joints (**c**) Region A presented at high magnification; (**d**) Region B presented at high magnification.

The fracture of the sample did not occur purely and neatly from the joint of the two materials. The shape of the sample was crisscrossed, and there was a mixed layer of brass and copper, Meanwhile, necking of the joint occurred. The fracture surface showed two areas at low magnification, and the specific morphology was observed under high magnification. Figure 7c,d shows a large number of dimples. The size and depths of the dimples in different parts were different, which showed that it was a coalescence mechanism of the micropores, the heterogeneity of the dimples was caused by the heterogeneous mixing of dissimilar materials, which was also the reason that mechanical properties of the joint were relatively low.

### 3.4. Microhardness and Intermetallic Compounds

**Figure 8:**
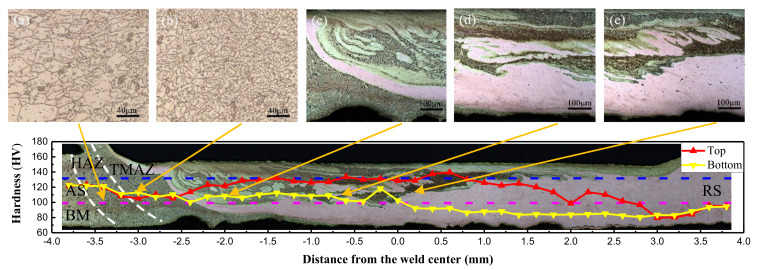
Hardness distribution characteristics of cross-section. (12,000 rpm, 280 mm/min) (**a**–**e**) are an enlarged view of the corresponding area.

**Figure 9:**
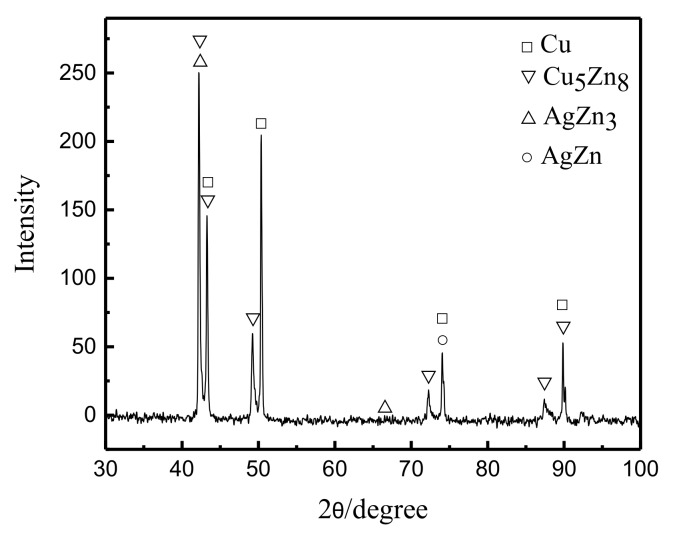
X-ray diffraction analysis of the weld zone. (12,000 rpm, 280 mm/min).

In the FSW process, the microhardness is also an important reflection of the fluidity of the metal. Figure 8 shows the microhardness distribution curve of the cross section of the joint under a rotation speed of 12,000 rpm and welding speed of 280 mm/min. Overall, there was a hardness gradient in the cross section of the joint, and the hardness of the upper layer was generally greater than that of the bottom. The hardness of the joint fluctuated up and down, and the hardness of the transition zone of the two metals changed abruptly. The larger value (brass) increased to the highest point and then decreased to a smaller value (copper). There were two main reasons for this phenomenon: The first reason was that the upper brass of the weld exhibited a large amount of migration, a more uniform distribution, dynamic recrystallization of the grains, and grain refinement caused by strong plastic deformation. The peak of hardness located at the right side of the weld center could reach 139.5 HV. Under the influence of the tool and the backing plates, the copper on the bottom metal migrated to the AS. The metal tended to flow upward and was widely distributed. Thus, its microhardness value was generally smaller, and its peak value was about 118.3 HV on the left side of the weld center. The second reason was that the hardness of the brass base was higher than that of copper, and metallurgical bonding occurred when two metals interweaved in the weld zone. Intermetallic compounds, Cu_5_Zn_8_, AgZn_3_, and AgZn were produced at the joint interface, which led to hardness fluctuations in the local area of the weld (Figure 9).

### 3.5. Collection and Analysis of Temperature, Axial Force, and Transverse Force During Welding Process

**Figure 10:**
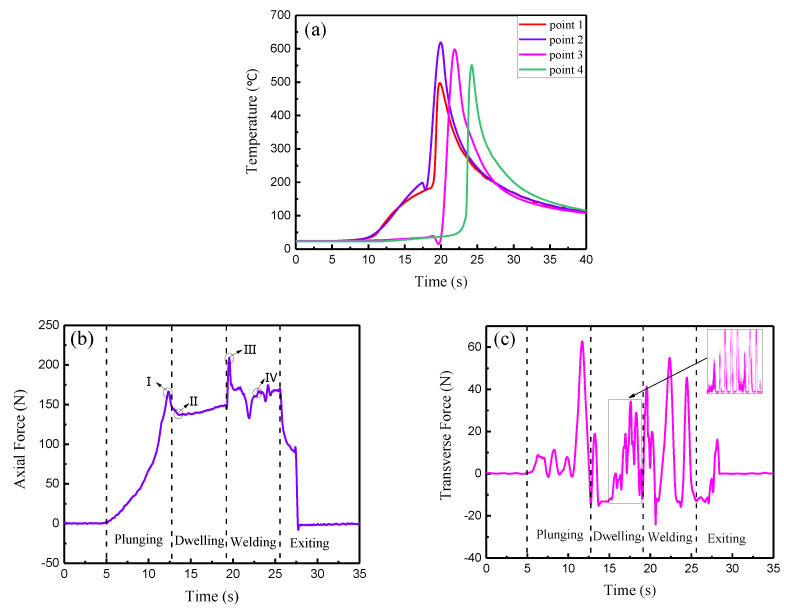
Dynamic change process of physical quantity during micro friction stir welding (μFSW) process: (**a**) dynamic temperature-time (**b**) axial force-time (**c**) transverse force-time.

The thermal cycling and stress strain behavior during the welding process determined the flow of the plastic metal and the properties of the joint. When the rotation speed was 12,000 rpm and the welding speed was 280 mm/min, the plastic metal flow of the joint was sufficient. Therefore, the temperature, axial force, and transverse force with these parameters were collected and analyzed to explore the dynamic variation process. The dynamic temperature curves are shown in Figure 10a. At the initial stage of welding, a large amount of heat was generated, and the weld temperature slowly increased with the increase in the contact area between the tool and the material. As the welding progressed, the friction heat and plastic deformation heat increased the rate of temperature increase. When the tool reached point 2, the temperature reached a maximum value of 618 °C, and at point 1 the temperature was about 497 °C. When the welding tended to a stable state, the temperature could reach up to 575 °C. At this time, the heat input and output of the weld reached a steady state, and the plasticized metal had better fluidity at this temperature. From the force time curves of the μFSW process (Figure 10b,c), based on the movement of the tool, the μFSW process could be divided into four stages, the plunging stage, the dwelling stage, the stable welding stage and the exiting stage. During the plunging stage, the low degree of material softening was due to less heat being generated by friction. The axial force increased rapidly with the slow plunging of the tool, and when the shoulder touched the base material surface, it reached a maximum value of 166 N (Figure 10(bI)). The softening amount of the base metal then increased with the increase in the heat input per unit time, and the axial force began to decrease slowly. When the plunge depth of the shoulder reached 0.06 mm, the dwelling stage began. The axial force rapidly decreased to a lower level of 137 N (Figure 10(bII)) due to the disappearance of the reaction force, and the metal softened to the plasticizing state. In the initial welding stage, the axial force suddenly increased to 209 N (Figure 10(bIII)), after which it began to drop rapidly to a lower level of 163 N (Figure 10(bIV)). Finally, when the tool rose and left the sheet surface, the whole welding process was finished, and the axial force dropped sharply toward 0 N. During the welding process, the directions of the frictional forces on the advancing and RSs was opposite and the physical properties of the metals were different, at the same time, there was a significant difference in the amount of softening and the degree of plasticization, which led to a large fluctuation of the transverse force at the 0 N. The positive value was much larger than the negative value. In the stable welding stage, the transverse force exhibited small fluctuations (Figure 10c).

### 3.6. Establishment of Plastic Metal Flow Model for Cross Section of Weld

**Figure 11:**
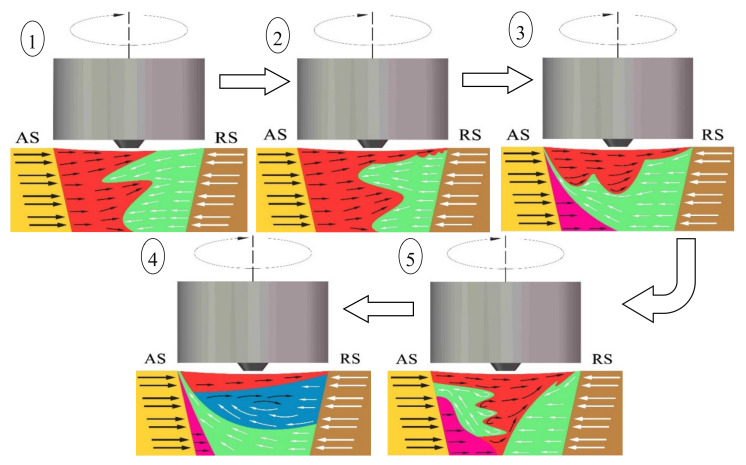
Model drawing of material flow on the cross-section.

During FSW processing, the interaction between the tool and the base material is accompanied by a complex process of energy consumption, transformation, and transfer. There are many factors that affect the flow of the plastic metal in the weld. In addition to conventional parameters, such as the rotation speed, welding speed and inclination angle, the tool profile, properties of the material itself, and change of the temperature field also play important roles in the formation of the weld. To analyze the material flow accurately, and understand the material migration behavior, a material flow model in the cross section of the weld was established. As shown in Figure 11, under different process parameters, the model of the weld metal plasticity flow from poor to good was as follows: ①~⑤ models. The whole weld was wide and narrow from top to bottom, with a trapezoidal distribution. The tool rotated in the clockwise direction, and the butt material was a dissimilar metal. The base materials are indicated by yellow (AS) and brown (RS), and the black and white arrows represent the metal migration path and direction. With the change of the material flow pattern, the area corresponding to the flow characteristics in the nugget zone also changed. The weld area could be divided into four regions: the shoulder influence area, the tool pin influence area, the weld bottom area, and the AS thermo-mechanically affecting area. Different colors were used in each area to represent the material flow.

The formation mechanism of the FSW joint of the copper/copper alloy was examined. Affected by the shoulder of the tool, the tool pin of the cone, and welding parameters, the material flow capacities of each area in the weld were significantly different. These areas were separated by clear interfaces in the cross section. When the ratio of the rotation speed to welding speed and the energy of the welding line were small, the metal migration occurred quickly in the action area of the shoulder and tool pin. In the shoulder influence area, the heat input was high, and the temperature quickly increased. The nearby metals were easily softened and reached a plastic state. Meanwhile, the linear velocity was large, and the plastic metal migrated from the AS to the RS, displaying a morphology with fluid-like and lamellar patterns. In the tool pin influence area, when the friction shear stress between the tool and the base material exceeded the inherent yielding shear stress, the metal began to undergo plastic yield and plastic deformation. The metal then flowed with the rotation of the tool, accompanied by a large amount of plastic deformation heat and frictional heat, displaying a morphology with lamellar patterns, corresponding to ① and ② in the model. When the ratio of the rotation speed to the welding speed increased, the heat input per unit time increased, the temperature gradient of the cross section of the weld decreased, and the metal at the bottom of the weld softened and plasticized. However, the dissimilar metals had different yield shear stresses and significant differences in flow capacity. Thus, under the constraints of the tool and the backing plate, the copper on the RS of the bottom of the weld migrated, and when encountering the resistance of the unplasticized metal, the copper began to migrate upward on the AS, forming a river-like structure. In the heat engine-affected zone on the AS, the temperature was lower, the stress was smaller, and excessive metal migration did not occur, as shown in models ③ and ④. The metal migration direction on the RS of the thick plate was the same as the rotating shear direction of the tool, and only a small amount of rotational flow migration occurred; When the ratio of the rotation speed to the welding speed was appropriate, the two plastic metals constantly flowed and intertwined with each other, a concentric vortex flow formed in a clockwise direction with a vortex-like structure, and physical and chemical processes, such as diffusion and recrystallization, occurred on the contact surface, generating intermetallic compounds in model ⑤. In general, plastic metal flows are characterized by randomness, diffusivity, and vorticity, and the flow speeds and directions are constantly changing. The plastic flow in the upper layer of the joint is usually greater than that in the bottom layer and flows along the horizontal direction. The middle area of the joint forms a vortex-like mixed structure, the difference in the capacities of the dissimilar metals causes them to flow, and the amount of metal moving from the RS to the AS of the bottom increases. However, plastic metal accumulation occurs. The mixed interlaced state of the material grain changes with the change in the welding parameters, reflecting the material flow under the action of the velocity field.

## 4. Conclusions

The main results of this investigation are as follows:

1. Pure copper (T2) and brass(H62) thin sheets were successfully joined by μFSW. When the rotation speed was 12,000 rpm and the welding speed was 280 mm/min, the material flow pattern was the best under the combined action of the thermal and force fields.
2. The dynamic physical quantity of welding is analyzed. Temperature, axial force and transverse force will increase to some extent, and then tend to dynamic balance to meet the requirements of welding.
3. In the metal flow mode, the process parameters that the thin plate offers play a greater role than the geometry of the tool, and the metal flow is more sufficient under the conditions of high rotation speed and low welding speed.

## Figures and Tables

**Table 1:** Chemical composition of the base materials. (mass fraction/%).

Element	Cu	Fe	Pb	S	Sb	As	Bi	Zn	Impurities
T2	99.9	0.005	0.005	0.005	0.002	0.002	0.001	—	—
H62	60.5~63.5	0.15	0.08	—	—	—	—	rest	0.5

**Table 2:** Physical and mechanical properties of the material.

Material	Density ρ/(g·cm^−3^)	Melting Point T/m(℃)	Thermal Conductivity λ/(W·m^−1^·K^−1^)	Elongation A/(%)	Tensile Strength /(MPa)	Hardness (HV)
T2	8.94	1083	391	6	235	92
H62	8.43	935	123	3	400	121

**Table 3:** Welding parameters.

Materials	Rotation Speed (rpm)	Welding Speed (mm/min)	Plunging Rate (mm/min)
H62/T2	10,000–14,000	240	6
	12,000	160–320	
